# Predictors of treatment response to semaglutide for weight management

**DOI:** 10.3389/fendo.2026.1893511

**Published:** 2026-09-17

**Authors:** Ploutarchos Tzoulis

**Affiliations:** Research Department of Experimental and Translational Medicine, University College London, London, United Kingdom

**Keywords:** antiobesity agent, GLP-1 agonist, obesity, semaglutide, weight loss

## Abstract

The emergence of weekly, injectable glucagon-like peptide-1 (GLP-1) receptor agonists has revolutionized the obesity management landscape. Numerous studies have shown high efficacy as well as great variability in weight loss, highlighting the need for this mini review, summarizing current evidence about predictors of semaglutide-related weight loss. The first well-known determinant of semaglutide-induced weight loss is the presence of type 2 DM which is associated with significantly lower body weight reduction. The second predictor is sex, with females exhibiting a greater weight loss than males by a mean 6%. In addition, higher maintenance dose of 7.2 mg is superior to standard 2.4 mg dose in weight reduction by a mean 3%. There is no clear association of age and baseline body mass index with the magnitude of semaglutide-driven weight loss, except for the possibility of adolescence enhancing and overweight reducing this treatment effect. The potential impact of genetic polymorphisms of individual treatment response to semaglutide remains incompletely understood, limited so far to a modest effect of specific variants in GLP1 receptor gene. Another potential predictor of response could be obesity phenotype, with preliminary data supporting greater effectiveness of semaglutide in individuals with ‘‘hungry gut’’ phenotype. Type of lifestyle modification does not seem to have a significant effect on the variability of semaglutide-driven weight loss. Further studies are needed to expand knowledge about predictors of response to semaglutide, paving the way for validated predictive models in order to guide clinical decision-making and provision of personalized obesity care.

## Introduction

Over the last three decades, the worldwide prevalence of obesity has more than doubled, rendering the obesity epidemic one of the most high-burden, high-cost public health issues across the globe (1). The emergence of highly efficacious glucagon-like peptide-1 (GLP-1) receptor agonists and their widespread use in clinical practice have led to a decline in the obesity rates (2). In December 2025, the World Health Organization (WHO) issued a global call towards a complete transformation of the approach to people living with obesity, suggesting a central role for modern pharmacotherapy combined with intensive lifestyle modification (3). This paradigm shift in obesity care brings new challenges since current drug capacity can cover less than 10% of people with obesity, posing a significant financial burden on healthcare systems and potentially exacerbating health disparities (1). Until incremental expansion of capacity, funding and access is reached, an equitable, transparent evidence-based framework is urgently needed to enable fair prioritisation of people living with obesity for prescription of GLP-1 agonists (3) and to ensure that these medications will reach those who need them most and would benefit most (1).

Several randomized clinical trials (RCTs) have demonstrated the great efficacy of injectable weekly semaglutide for weight management, with a mean weight loss of 15-16%, but they have also reported great variability in the magnitude of weight reduction, including at the one end of the spectrum 10-13% of participants not reaching the 5% weight reduction threshold and at the other end of the spectrum 32-38% achieving more than 20% weight loss (4, 5). This significant heterogeneity in medication-induced weight loss also applies to tirzepatide, a dual agonist of GLP-1 and glucose-dependent insulinotropic polypeptide (GIP) (6). For these reasons, there has been great interest in identifying predictors of treatment effect, guiding personalized clinical decision-making. In view of the limited evidence base about predicting response to tirzepatide, this mini review will focus on summarizing scientific literature about predictors of response to semaglutide.

## Methods

We undertook a search via *PubMed* and the Cochrane Library until 28^th^ May 2026 of all publications, including the key words ‘GLP-1 agonists’, ‘glucagon-like peptide-1 receptor agonists’, ‘incretin mimetics’, ‘semaglutide’, ‘tirzepatide’, ‘antiobesity medications’, ‘pharmacotherapy for obesity’, and ‘medications for weight loss’. Data from various types of studies, ranging from real-world observations to RCTs and meta-analyses, were taken into consideration. The primary endpoint chosen for the assessment of treatment response and its relationship with different parameters was the total percentage weight loss over the duration of the study period. However, studies using different primary outcomes, such as percentage of participants reaching a specific threshold of weight reduction, were also considered.

### Predictors of response to semaglutide

#### Glycaemic status

Semaglutide therapy leads to significantly lower weight loss in individuals with type 2 Diabetes Mellitus (DM) compared to those without, as evidenced by a mean 68-week reduction in bodyweight of 9.6% (7) versus 14.9% (4), respectively. Interestingly, it remains unclear whether this association extends across the continuum of glucose levels to the presence of prediabetes. On the one hand, in STEP 1 trial extension, those with prediabetes had numerically lower decrease in body weight of 15.7% versus 19.7% in those with normoglycemia (8), but on the other hand, 4-year weight loss in semaglutide-treated participants in the SELECT trial was similar in those with prediabetes and normoglycemia (9). In addition, STEP 10 trial, evaluating semaglutide in people with obesity and prediabetes, showed a mean weight reduction of 13.9% (10), similar with that reported in studies including people with obesity and either normoglycemia or prediabetes (4). Taking into account all these data, the impact of prediabetes at baseline on the magnitude of weight loss has not been clarified yet. It’s worth mentioning that confounding may be introduced because of indirect comparison between studies with different populations and, also, that prediabetes is an umbrella term covering a spectrum, including impaired fasting glycemia, impaired glucose tolerance and their combination. Amongst semaglutide-treated patients with type 2 DM, higher baseline glycated haemoglobin (HbA1c), longer duration of DM, concomitant insulin administration and larger number of antidiabetic medications have been associated with inferior weight loss (11).

Therefore, the presence or absence of type 2 DM has been the most studied predictor of semaglutide-related weight loss, with individuals with type 2 DM achieving significantly lower weight reduction than those without. Studies are needed to evaluate whether baseline HbA1c or fasting glucose or markers of insulin resistance, such as Homeostatic Model Assessment of Insulin Resistance (HOMA-IR), are associated with the extent of semaglutide-induced weight loss across the continuum of normoglycemia and prediabetes.

#### Sex

There seems to be sex-specific differences in the efficacy of semaglutide for the treatment of obesity, with STEP studies reporting a mean weight reduction of 14.0-16.2% in women and 8.0-9.3% in men (12), accounting for a greater weight loss by a mean 6% among females than among males (13). Two recent meta-analyses of trials have also shown a greater weight loss in women than men for five different GLP-1 agonists, highlighting sex as a major differentiator (14).

A growing body of evidence supports sex differences in response to GLP-1 analogues, highlighting the key role of oestrogens (15). Multiple preclinical studies have shown (i) the synergistic interaction of GLP-1 and its analogues with oestrogens, (ii) that oestrogens enhance the effect of GLP-1 and its suppressive effect on food-motivated behaviour, (iii) that the exogenous addition or removal of oestrogens influences the effects of GLP-1 analogues on feeding behaviour, and (iv) that the presence of oestrogens leads to activation of additional brain areas and downstream signals which otherwise remain unaffected (15). The enhanced response of women might also be explained to some extent by exposure difference due to lower starting weight in females than males. Moreover, women exhibit slower gastric emptying compared to males, while a significant proportion of men develop excess adiposity-related hypogonadotropic hypogonadism which may render obesity resistant to treatment (12). Data are still lacking about the treatment response to semaglutide in women before and after menopause as well as the impact of Hormone Replacement Therapy (HRT) and any other treatment affecting reproductive hormones on the weight loss outcomes. In total, both preclinical and clinical data confirm the key contribution of gonadal steroids to the enhanced response of females to incretins (15), leading to greater weight loss in females than males.

#### Maintenance dose

Two recently published RCTs, STEP UP and STEP UP T2D trials, undertaking head-to-head comparison of different maintenance semaglutide doses in terms of weight loss outcomes, have reported superior weight loss with 7.2 mg semaglutide dose compared to standard 2.4 mg maintenance dose by a mean 3% in people with obesity, without and with type 2 DM (16, 17). These findings suggest that semaglutide-induced weight loss is dose-dependent, while the rates of discontinuation and severe adverse events was similar with the exception for more frequent occurrence of dysesthesia in those receiving semaglutide at 7.2 mg dose (16, 17).

#### Age and baseline BMI

A systematic review has not determined any significant effect of age and baseline body mass index (BMI) on treatment response to GLP-1 analogues (14). However, an analysis of SELECT trial, a 4-year cardiovascular outcomes trial of semaglutide-treated people with excess weight, reported a linear relationship between age group and degree of weight reduction, with younger age being associated with greater weight loss (9). Of note, the actual observed difference was small, with mean weight loss percentage of 9.3% in people under the age of 55 years compared to 7.5% in individuals aged above 75 years old (9). Another interesting observation was that people with baseline BMI < 30 kg/m^2^ achieved a mean percentage weight loss of 7.5% compared to 9.2% in participants with BMI > 40 kg/m^2^, while weight loss was similar across different classes of obesity. The reason behind this observation might be that individuals with overweight are closer to their ‘‘set point’’ where the weight loss reaches a plateau, but this is a hypothesis awaiting to be tested (9). Examining the efficacy of semaglutide as antiobesity medication (AOM) across different age groups, semaglutide-treated adolescents achieved a significant placebo-extracted reduction in body weight, 5% greater than adults (18). At the other end of the age spectrum, a pooled analysis of SUSTAIN 1–5 trials has showed a similar weight loss in older semaglutide-treated patients with type 2 DM with those younger than 65 years (19). A meta-analysis of five studies assessing GLP-1 agonists in patients with obesity found that people older than 65 years old experience similar weight loss with younger individuals (20).

In conclusion, the effect of age and BMI subgroup on treatment effect of semaglutide is limited, if any, with two possible exceptions. Firstly, adolescence may enhance its treatment effect (18), but this pronounced effect needs to be reproduced in large-scale studies. In this case, it remains to be determined whether this association extends to those younger than 30 years old. Second, people with overweight may experience smaller percentage of body weight reduction than those with obesity.

#### Genetic factors

The glycaemic response to GLP-1 agonists is associated with the presence of single nucleotide polymorphisms (SNPs), especially in the GLP1 receptor gene (21). Until recently, pharmacogenomic studies had failed to identify consistent genome-wide associations for incretin-induced weight loss (22, 23). However, a recent large-scale genome-wide association study (GWAS) identified a significant association of a missense variant rs10305420 in GLP1R gene with enhanced efficacy of both semaglutide and tirzepatide, as evidenced by a mean additional weight loss of 0.76 kg (24). This association was statistically significant, albeit of questionable clinical significance due to the small effect size.

The potential contribution of genetic factors to individual treatment response remains incompletely understood, highlighting the need for future studies evaluating the predictive utility of genetic factors (24).

#### Obesity phenotypes

A phenotype-guided treatment approach of people with obesity has been proposed, with preliminary data supporting that individuals with ‘‘hungry gut’’ phenotype, characterized by abnormal satiety and evaluated by the rate of gastric emptying and the duration of fullness after a meal, are the best responders to GLP-1 receptor agonists (25). However, application of obesity phenotyping in clinical practice can be cumbersome and time-consuming, with the availability of required tools being limited to research settings. For this reason, a machine-learning- assisted genetic risk score (GRS) was developed to predict high or low CTS (calories to satiation), the number of calories needed for a person to reach satiation, with those having low CTS being classified as ‘‘hungry gut’’ (26). Preliminary data have shown significantly greater weight loss outcomes in individuals with a GRS indicative of low CTS compared to those with high CTS, for both liraglutide (26) and semaglutide. Based on these data, MyPhenome test is currently available in the United States as a machine-learning, genetic-based test. However, large-scale prospective studies are warranted to evaluate the reproducibility of preliminary findings, its generalizability to different populations, its predictive power for treatment response to different AOMs, and its cost-effectiveness.

A subgroup of people with obesity is characterized by higher levels of anxiety, emotional eating, cravings and reward-seeking behaviour, classified as having the ‘‘emotional hunger’’ phenotype (25). Our hypothesis which remains to be tested is that these emotional traits may to some extent counteract the physiological action of increased GLP-1 levels, resulting in diminished efficacy of incretin mimetics (25). On the one hand, small studies have demonstrated positive effect of both semaglutide and tirzepatide on emotional eating, cravings, and other abnormal eating behavioural patterns (27, 28), while real-world data support that semaglutide leads to significant reduction of ‘‘food noise’’, defined as a constant, obsessive and intrusive stream of food-related thoughts which is distinct of physiological sense of hunger. On the other hand, an observational study of people with type 2 DM treated with GLP-1 agonist showed sustained suppression of external eating scores in response to external cues, but less pronounced effect on emotional eating scores, indicating emotional eating as a potential predictor of reduced responsiveness to GLP-1 agonists (29). In total, the efficacy of semaglutide in people with ‘‘emotional hunger’’ phenotype warrants further study.

#### Type of lifestyle modification

Among semaglutide-treated individuals, the addition of intensive behavioural therapy combined with low-calorie, meal-replacement diet (5) did not lead to greater long-term weight loss compared to less intensive lifestyle intervention (4), indicating a neutral effect of highly intensive lifestyle interventions on the magnitude of overall weight loss (5).Data are scarce about the effect of different medical nutrition therapies and physical exercise on the magnitude of weight loss in semaglutide-treated individuals. So far, studies of pharmacotherapy have focused on the efficacy and safety of AOMs as an adjunct to standard lifestyle measures. In light of paucity of data, future studies are needed to evaluate the impact of different dietary and exercise regimens not only on the efficacy and tolerability of AOMs, but also on body composition and sustainability of weight loss after drug discontinuation.

The key emerging role of dietary intervention is that of minimizing loss of skeletal muscle and optimizing body composition combined with tackling weight regain after treatment discontinuation (30). In a similar manner, physical exercise has indisputable efficacy for preventing or, at least, reducing weight regain following semaglutide discontinuation (31), while resistance exercise training is widely recommended to maintain muscle mass and function (32, 33).

#### Psychiatric disease

The reciprocal link between mental health illness and excess adiposity raises the question whether the presence of psychiatric disease affects treatment response to GLP-1 analogues. Two small RCTs of antipsychotic-treated patients with schizophrenia and obesity have demonstrated great efficacy of semaglutide for weight loss (34, 35). However, both studies had small sample size, limited duration 30–38 weeks and lower than commonly used maintenance doses of 1.0-2.0 mg. Therefore, the semaglutide-induced weight loss outcomes in patients with schizophrenia receiving antipsychotic medications are not comparable to the findings of other studies. As far as active major depressive disorder (MDD) is concerned, STEP trials have excluded patients with active MDD or other severe psychiatric condition (36). A *post hoc* subgroup analysis of STEP trials has demonstrated that the use of antidepressants medications for other indications than depression does not alter the treatment response to semaglutide (36). The only data supporting a possibly limited semaglutide efficacy in people with active major depressive disorder (MDD) were reported in a small, non-controlled, real-world, retrospective study with several potential confounders, such as questionable compliance or early discontinuation for various reasons other patients (37). Therefore, the efficacy of GLP-1 analogues in people with obesity and active depression remains unknown and warrants evaluation in prospective studies.

## Discussion

The main determinants of the magnitude of semaglutide-induced weight loss are the presence or absence of type 2 DM and sex. Twelve-month semaglutide treatment at the 2.4 mg weekly maintenance dose leads to a mean weight loss of 14-18% in females and 8-11% in males without type 2 DM, while the mean body weight reduction is diminished in people with type 2 DM, to 7.5% and 4.6% in women and men, respectively (12, 13).

At present, a ‘‘trial and error’’ approach is followed in prescribing pharmacotherapy for obesity, with the early treatment response at various time points, such as week 8, week 16 (8, 38), and week 24 (39) predicting individual weight loss trajectories (38, 39). Entering the era of precision medicine, a model was recently validated to predict the glucose-lowering effect of various drug classes in patients with type 2 DM (40). However, the lack of robust evidence basis does not currently allow the development of a similar multivariable model for the prediction of treatment response to AOMs, as summarized in **Table 1**. Nowadays, only three predictors of treatment response to semaglutide have been established; (i) presence or absence of type 2 DM, (ii) sex, and (iii) maintenance dose (1 mg or 2.4 mg or 7.2 mg) characterized by a clear dose-response relationship, with higher semaglutide doses leading to greater amounts of weight loss (41, 16). Age and BMI, across a continuum, do not make a significant contribution to treatment effect. However, upon confirmation of preliminary data, younger age and overweight are potential enhancer and reducer of semaglutide response, respectively. The role of genetic polymorphisms hasn’t been fully understood yet, with the contribution of variants to GLP1 receptor gene being modest at best, currently excluding them from a prediction model. The obesity phenotype is also emerging as a possible determinant of the AOM selection in the future. At present, scarce data support the use of a genetic risk score, according to which those with low ‘‘calories to satiation’’ score exhibit exaggerated response to semaglutide, with these findings requiring confirmation in large-scale prospective studies. In addition, studies are required to evaluate the effect of the type of medical nutrition therapy or physical exercise prescription on the magnitude of weight loss in people receiving AOMs. Finally, the presence of schizophrenia plays a neutral role on the effect to semaglutide, while the likely impact of active major depression remains be studied. Future model predictors would incorporate clinical and genetic data, with potential response enhancers being younger age, ‘‘hungry gut’’ phenotype, and presence of variant in GLP1R gene, while the role of obesity phenotyping for provision of individualised obesity care remains to be determined.

**Table 1:** Summary of the effect of various parameters on semaglutide-related weight loss outcomes.

Parameter	Weight loss	Effect size	Evidence
Type 2 DM	lower	by 5%	Indirect comparison of RCTs
Female sex	greater	by 6%	Post hoc RCT analysis
7.2 mg dose	greater	by 3%	Head-to-head within RCT
Age	unknown	unknown	Post hoc RCT analysis
BMI	unknown	unknown	Post hoc RCT analysis
GLP1R variant	greater	by 0.76 kg	Single GWAS
Low CTS phenotype	greater	by 3-4%	Single-centre, small RCT
Lifestyle	unknown	unknown	Lack of data
Schizophrenia	unknown	unknown	Indirect comparison of RCTs
Depression	unknown	unknown	Lack of data

Type 2 DM, Type 2 Diabetes mellitus; BMI, Body Mass Index; GLP1R, Glucagon-Like Peptide-1 Receptor; CTS, Calories To Satiation; RCTs, Randomized Controlled Trials; GWAS, Genome Wide Association Study.

In line with semaglutide, tirzepatide is also characterized by great variability in treatment effect, with 9% of tirzepatide-treated individuals not achieving a clinically meaningful weight loss in contrast to one third of people exceeding 25% weight loss (6). People with type 2 DM had a mean weight loss of 11.6% (42), significantly lower than that of 20.9% in those without (6), while females achieved greater weight loss than males by approximately 6% (13). A novel observation of a retrospective cohort study was the association of concurrent HRT with an additional 5% weight loss in postmenopausal women receiving tirzepatide, suggesting a potential synergistic effect of exogenous oestrogens and tirzepatide on weight loss outcomes.

In a rapidly evolving landscape of obesity pharmacotherapy, the armamentarium of gut hormones-based therapies in 2027 is expected to include the following classes of drugs: (i) injectable GLP-1 agonists, weekly semaglutide at a maintenance dose of either 2.4 mg or 7.2 mg and daily liraglutide, (ii) oral GLP-1 agonists, daily semaglutide and daily orforglipron, (iii) dual GLP1-GIP agonists, such as weekly injectable tirzepatide, (iv) dual GLP1-amylin agonist, named CagriSema, a weekly injectable combination of semaglutide and cagrilintide, and (v) triple GLP1-GIP-glucagon agonists, such as weekly injectable retatrutide (43). This new era of multiple effective pharmacotherapies with different mechanisms of action, routes of administration, efficacy-safety profiles, cost-effectiveness and different effects on various obesity-related complications highlights the need to develop multivariable models to predict treatment response to AOMs, enabling provision of personalized obesity care in day-to-day care (44).

Following the new care paradigm of personalized management in type 2 DM, the time has come to move beyond one-size-fits-all approach towards precision medicine for obesity. The great inter-person variability in the treatment effect of AOMs highlights the need for studies examining the impact of various demographic, metabolic, behavioural, and genetic factors on the treatment response to pharmacotherapy in people with obesity (44). Therefore, precision medicine initiatives, integrating data from genetics, epigenetics and metabolomics as well as encompassing demographic, environmental and psychological factors, are currently in progress in order to stratify obesity into different phenotypes with distinct biological basis, risk for future complications and treatment approach (45, 46). Despite significant scientific breakthroughs in the field of obesity, evidence to date does not support yet the application of genetics, nutrigenomics, pharmacogenetics, microbiomics, transcriptomics, and metabolomics on guiding the therapeutic strategy for each person with obesity (47). Finally, the rapidly increasing integration of artificial intelligence (AI) into healthcare suggests a possible future role of AI-driven models in provision of personalized obesity care.

In conclusion, there is a great heterogeneity in the individual response to semaglutide, while our predictive ability remains limited. Current evidence is inadequate to enable development of a prediction model since only two factors, glycaemic status and gender, have a significant effect on treatment response for a given dose of a medication. Future studies are urgently warranted to explore the impact of various biomarkers on treatment response, paving the way for validated predictive models and algorithms in order to guide clinical decision-making (45, 48) and enable clinicians to provide the right treatment for the right patient at the right time (45).

## References

[1] Schweitzer K . What to know about the WHO's new GLP-1 drug guideline. JAMA. (2026) 335(5):388–90. doi: 10.1001/jama.2025.25208 41511764

[2] Rader B Hazan R Brownstein JS . Changes in adult obesity trends in the US. JAMA Health Forum. (2024) 5:e243685. doi: 10.1001/jamahealthforum.2024.3685 39671205 PMC11645646

[3] Celletti F Farrar J De Regil L . World Health Organization guideline on the use and indications of glucagon-like peptide-1 therapies for the treatment of obesity in adults. JAMA. (2025) 335(5):434–8. doi: 10.1001/jama.2025.24288 41324410

[4] Wilding JPH Batterham RL Calanna S Davies M Van Gaal LF Lingvay I . Once-weekly semaglutide in adults with overweight or obesity. N Engl J Med. (2021) 384:989–1002. doi: 10.1056/nejmoa2032183 33567185

[5] Wadden TA Bailey TS Billings LK Davies M Frias JP Koroleva A . Effect of subcutaneous semaglutide vs placebo as an adjunct to intensive behavioral therapy on body weight in adults with overweight or obesity: The STEP 3 randomized clinical trial. JAMA. (2021) 325:1403–13. doi: 10.1001/jama.2021.1831 33625476 PMC7905697

[6] Jastreboff AM Aronne LJ Ahmad NN Wharton S Connery L Alves B . Tirzepatide once weekly for the treatment of obesity. N Engl J Med. (2022) 387:205–16. doi: 10.1056/nejmoa2206038 35658024

[7] Davies M Faerch L Jeppesen OK Pakseresht A Pedersed SD Perreault L . Semaglutide 2.4 mg once a week in adults with overweight or obesity, and type 2 diabetes (STEP 2): a randomised, double-blind, double-dummy, placebo-controlled, phase 3 trial. Lancet. (2021) 397:971–84. doi: 10.1016/s0140-6736(21)00213-0 33667417

[8] Wilding JPH Batterham RL Davies M Van Gaal LF Kandler K Konakli K . Weight regain and cardiometabolic effects after withdrawal of semaglutide: The STEP 1 trial extension. Diabetes Obes Metab. (2022) 24:1553–64. doi: 10.1111/dom.14725 35441470 PMC9542252

[9] Ryan DH Lingvay I Deanfield J Kahn SE Barros E Burguera B . Long-term weight loss effects of semaglutide in obesity without diabetes in the SELECT trial. Nat Med. (2024) 30:2049–57. doi: 10.1038/s41591-024-02996-7 38740993 PMC11271387

[10] McGowan BM Bruun JM Capehorn M Pedersen SD Pietilainen KH Muniraju HAK . Efficacy and safety of once-weekly semaglutide 2.4 mg versus placebo in people with obesity and prediabetes (STEP 10): a randomised, double-blind, placebo-controlled, multicentre phase 3 trial. Lancet Diabetes Endocrinol. (2024) 12:631–42. doi: 10.1016/s2213-8587(24)00182-7 39089293

[11] Ghusn W Anazco D Fansa S Tama E Cifuentes L Gala K . Weight loss outcomes with semaglutide based on diabetes severity using the individualized metabolic surgery score. EClinicalMedicine. (2024) 72:102625. doi: 10.1016/j.eclinm.2024.102625 38756106 PMC11096862

[12] Jensterle M Rizzo M Janez A . Semaglutide in obesity: Unmet needs in men. Diabetes Ther. (2023) 14:461–5. doi: 10.1007/s13300-022-01360-7 36609945 PMC9981825

[13] Aronne LJ Horn DB le Roux CW Ho W Falcon BL Gomez Valderas E . Tirzepatide as compared with semaglutide for the treatment of obesity. N Engl J Med. (2025) 393:26–36. doi: 10.1056/nejmoa2416394 40353578

[14] Alexander GC Xiao X Dilek S Lewis S Deng Q Kim M . Heterogeneity of treatment effects of glucagon-like peptide-1 receptor agonists for weight loss in adults: A systematic review and meta-analysis. JAMA Intern Med. (2026) 186(5):567–77. doi: 10.1001/jamainternmed.2025.8222 41770554 PMC12954602

[15] Borchers S Skibicka KP . GLP-1 and its analogs: Does sex matter? Endocrinology. (2025) 166(2):bqae165. doi: 10.1210/endocr/bqae165 PMC11733500 39715341

[16] Wharton S Freitas P Hjelmesaeth J Kabisch M Kandler K Lingvay I . Once-weekly semaglutide 7.2 mg in adults with obesity (STEP UP): a randomised, controlled, phase 3b trial. Lancet Diabetes Endocrinol. (2025) 13:949–63. doi: 10.1016/s2213-8587(25)00226-8 40961952

[17] Lingvay I Bergenheim SJ Buse JB Freitas B Garvey WT Harder-Lauridsen NM . Once-weekly semaglutide 7.2 mg in adults with obesity and type 2 diabetes (STEP UP T2D): a randomised, controlled, phase 3b trial. Lancet Diabetes Endocrinol. (2025) 13:935–48. doi: 10.1016/s2213-8587(25)00225-6 40961953

[18] Weghuber D Barrett T Barrientos-Perez M Gies I Hesse D Jeppesen OK . Once-weekly semaglutide in adolescents with obesity. N Engl J Med. (2022) 387:2245–57. doi: 10.1530/ey.20.9.17 36322838 PMC9997064

[19] Warren M Chaykin L Trachtenbarg D Nayak G Wijayasinghe N Cariou B . Semaglutide as a therapeutic option for elderly patients with type 2 diabetes: Pooled analysis of the SUSTAIN 1-5 trials. Diabetes Obes Metab. (2018) 20:2291–7. doi: 10.1111/dom.13331 29687620 PMC6099273

[20] Rego de Figueiredo I Simas FS Ghiletchi A Oliveira Torres J Silva-Nunes J . Safety and efficacy of glucagon-like peptide-1 receptor agonists use in elderly people with obesity-a meta-analysis. Obes (Silver Spring). (2026). doi: 10.1002/oby.70098 41640092 PMC13535706

[21] Dawed AY Mari A Brown A McDonald TJ Li L Wang S . Pharmacogenomics of GLP-1 receptor agonists: a genome-wide analysis of observational data and large randomised controlled trials. Lancet Diabetes Endocrinol. (2023) 11:33–41. doi: 10.1016/s2213-8587(22)00340-0 36528349

[22] German J Cordioli M Tozzo V Urbut S Arumae K Smir RAJ . Association between plausible genetic factors and weight loss from GLP1-RA and bariatric surgery. Nat Med. (2025) 31:2269–76. doi: 10.1038/s41591-025-03645-3 40251273 PMC12283387

[23] Apostolopoulou M Koufakis T . Predicting treatment response to GLP-1 receptor agonists: still tossing the coin or doing better? Expert Opin Pharmacother. (2025) 26:1113–5. doi: 10.1080/14656566.2025.2517802 40476563

[24] Su QJ Ashenhurst JR Xu W Tran V Ryanne Wu R Weldon CH . Genetic predictors of GLP1 receptor agonist weight loss and side effects. Nature. (2026) 653(8115):770–5. doi: 10.1038/s41586-026-10330-z 41951734 PMC13190316

[25] Acosta A Camilleri M Abu Dayyeh B Calderon G Gonzalez D McRae A . Selection of antiobesity medications based on phenotypes enhances weight loss: A pragmatic trial in an obesity clinic. Obes (Silver Spring). (2021) 29:662–71. doi: 10.1002/oby.23120 33759389 PMC8168710

[26] Cifuentes L Anazco D O'Connor T Hurtado MD Ghusn W Campos A . Genetic and physiological insights into satiation variability predict responses to obesity treatment. Cell Metab. (2025) 37:1655–66:e5. doi: 10.1016/j.cmet.2025.05.008 40482646 PMC12236147

[27] Nicolau J Pujol A Tofe S Bonet A Gil A . Short term effects of semaglutide on emotional eating and other abnormal eating patterns among subjects living with obesity. Physiol Behav. (2022) 257:113967. doi: 10.1016/j.physbeh.2022.113967 36162525

[28] Martin CK Carmichael OT Carnell S Considine RV Kareken DA Dydak U . Tirzepatide on ingestive behavior in adults with overweight or obesity: a randomized 6-week phase 1 trial. Nat Med. (2025) 31:3141–50. doi: 10.1038/s41591-025-03774-9 40555748 PMC12443625

[29] Koide Y Kato T Hayashi M Daido M Maruyama T Ishihara T . Association between eating behavior patterns and the therapeutic efficacy of GLP-1 receptor agonists in individuals with type 2 diabetes: a multicenter prospective observational study. Front Clin Diabetes Healthc. (2025) 6:1638681. doi: 10.3389/fcdhc.2025.1638681 41040428 PMC12483915

[30] Wadden TA Chao AM Moore M Tronieri JS Gilden A Amaro A . The role of lifestyle modification with second-generation anti-obesity medications: Comparisons, questions, and clinical opportunities. Curr Obes Rep. (2023) 12:453–73. doi: 10.1007/s13679-023-00534-z 38041774 PMC10748770

[31] Rubino D Abrahamsson N Davies M Hesse D Greenway FL Jensen C . Effect of continued weekly subcutaneous semaglutide vs placebo on weight loss maintenance in adults with overweight or obesity: The STEP 4 randomized clinical trial. JAMA. (2021) 325:1414–25. doi: 10.1001/jama.2021.3224 33755728 PMC7988425

[32] Locatelli JC Costa JG Haynes A Naylor LH Fegan PG Yeap BB . Incretin-based weight loss pharmacotherapy: Can resistance exercise optimize changes in body composition? Diabetes Care. (2024) 47:1718–30. doi: 10.2337/figshare.25483273.v1 38687506

[33] Lieberman DE Aslan DH Heymsfield SB . The conundrum of exercise for weight management in the GLP-1 receptor agonist era. JAMA. (2026) 335(21):1841–3. doi: 10.1001/jama.2026.5537 42081217

[34] Siskind D Baker A Arnautovska U Warren N Russell A DeMonte V . Efficacy and safety of semaglutide versus placebo for people with schizophrenia on clozapine with obesity (COaST): a phase 2, multi-centre, participant and investigator- blinded, randomised controlled trial in Australia. Lancet Psychiatry. (2025) 12:493–503. doi: 10.1016/s2215-0366(25)00129-4 40506208

[35] Ganeshalingam AA Uhrenholt N Arnfred S Gaede P During S Stenager EN . Semaglutide treatment of antipsychotic-treated patients with schizophrenia, prediabetes, and obesity: The HISTORI randomized clinical trial. JAMA Psychiatry. (2025) 82:1065–74. doi: 10.1001/jamapsychiatry.2025.2332 40900607 PMC12409653

[36] Kushner RF Fink-Jensen A Frenkel O McGowan B Goldman B Overvad M . Efficacy and safety of semaglutide 2.4 mg according to antidepressant use at baseline: A post hoc subgroup analysis. Obes (Silver Spring). (2024) 32(2):273–80. doi: 10.1002/oby.23946 37989717

[37] Tzoulis P Batavanis M Baldeweg S . A real-world study of the effectiveness and safety of semaglutide for weight loss. Cureus. (2024) 16:e59558. doi: 10.1530/endoabs.86.p86 38826889 PMC11144277

[38] Strathe A Horn DB Larsen MS Rubino D Sorrig R Tran MTD . A model-based approach to predict individual weight loss with semaglutide in people with overweight or obesity. Diabetes Obes Metab. (2023) 25:3171–80. doi: 10.1111/dom.15211 37424165

[39] Aronne LJ Horn DB Kokkinos AD Falcon BL Wang H Hoffmann Ht . Relationship of early rapid weight loss to efficacy and safety of tirzepatide and semaglutide for obesity: SURMOUNT-5 post hoc analysis. Am J Med. (2026) 139(7):913–21. doi: 10.1016/j.amjmed.2026.03.010 41865857

[40] Dennis JM Young KG Cardoso P Gudemann LM McGovern AP Farmer A . A five-drug class model using routinely available clinical features to optimise prescribing in type 2 diabetes: a prediction model development and validation study. Lancet. (2025) 405:701–14. doi: 10.1016/s0140-6736(24)02617-5 40020703

[41] O'Neil PM Birkenfeld AL McGowan B Mosenzon O Pedersen SD Wharton S . Efficacy and safety of semaglutide compared with liraglutide and placebo for weight loss in patients with obesity: a randomised, double-blind, placebo and active controlled, dose-ranging, phase 2 trial. Lancet. (2018) 392(10148):637–49. doi: 10.1016/S0140-6736(18)31773-2 30122305

[42] Garvey WT Frias JP Jastreboff AM Le Roux CW Sattar N Aizenberg D . Tirzepatide once weekly for the treatment of obesity in people with type 2 diabetes (SURMOUNT-2): a double-blind, randomised, multicentre, placebo-controlled, phase 3 trial. Lancet. (2023) 402:613–26. doi: 10.1016/s0140-6736(23)01200-x 37385275

[43] Melson E Ashraf U Papamargaritis D Davies MJ . What is the pipeline for future medications for obesity? Int J Obes (Lond). (2025) 49:433–51. doi: 10.1038/s41366-024-01473-y 38302593 PMC11971045

[44] Tzoulis P Baldeweg SE . Semaglutide for weight loss: unanswered questions. Front Endocrinol (Lausanne). (2024) 15:1382814. doi: 10.3389/fendo.2024.1382814 38904050 PMC11188346

[45] Tahrani AA Panova-Noeva M Schloot NC Hennige AM Soderberg J Nadglowski J . Stratification of obesity phenotypes to optimize future therapy (SOPHIA). Expert Rev Gastroenterol Hepatol. (2023) 17(10):1031–9. doi: 10.1080/17474124.2023.2264783 37791820

[46] Abraham A Yaghootkar H . Identifying obesity subtypes: A review of studies utilising clinical biomarkers and genetic data. Diabetes Med. (2023) 40:e15226. doi: 10.1111/dme.15226 37704218

[47] Cifuentes L Hurtado AM Eckel-Passow J Acosta A . Precision medicine for obesity. Dig Dis Interv. (2021) 5:239–48. doi: 10.1055/s-0041-1729945 36203650 PMC9534386

[48] Bomberg EM Ryder JR Brundage RC Straka RJ Fox CK Gross AC . Precision medicine in adult and pediatric obesity: a clinical perspective. Ther Adv Endocrinol Metab. (2019) 10:2042018819863022. doi: 10.1177/2042018819863022 31384417 PMC6661805

